# Associations Between Baseline Biomarkers and Functional Outcomes in Patients With Diabetic Macular Edema After Anti‐VEGF Treatment

**DOI:** 10.1155/joph/5243432

**Published:** 2026-09-02

**Authors:** Yi Cai, Lina Lv, Ziyi Wei, Yi Gao, Zeming Wu, Yanan Wang, Yong Liu, Rongrong Li

**Affiliations:** ^1^ Hebei Eye Hospital, Xingtai, Hebei, China; ^2^ Hebei Provincial Key Laboratory of Ophthalmology, Xingtai, Hebei, China; ^3^ Hebei Provincial Clinical Research Center for Eye Diseases, Xingtai, Hebei, China

**Keywords:** anti-VEGF, best-corrected visual acuity, diabetic macular edema, multifocal electroretinography, spectral domain optical coherence tomography

## Abstract

**Objective:**

To investigate associations between baseline structural and functional biomarkers and 6‐month post‐treatment best‐corrected visual acuity (BCVA) among patients with diabetic macular edema (DME) receiving intravitreal anti‐VEGF therapy.

**Methods:**

Data from patients treated at Hebei Eye Hospital between December 2022 and January 2024 were retrospectively analyzed. Baseline biomarkers, including the disruption of the external limiting membrane (ELM) and ellipsoid zone (EZ), disorganization of retinal inner layers (DRIL), and epiretinal membrane (ERM) on spectral domain optical coherence tomography (SD‐OCT), as well as the amplitude and implicit time of N1 and P1 waves in multifocal electroretinography (mfERG), were collected. The associations between these biomarkers and BCVA after anti‐VEGF treatment were then assessed. Linear regression analysis was applied to quantify the correlations between baseline SD‐OCT morphological biomarkers and 6‐month anti‐VEGF visual therapeutic efficacy. Multivariate logistic regression analysis was conducted to assess the efficacy of anti‐VEGF treatment and baseline mfERG biomarkers.

**Results:**

A total of 87 eligible DME patients completed three monthly anti‐VEGF loading injections and 6‐month follow‐up, including 48 patients in the response group and 39 in the nonresponse group. Linear regression assessed whether baseline retinal layer disruption predicted 6‐month visual response and revealed that extensive ELM (*t* = 2.914, *p* = 0.004) and EZ (*t* = 2.112, *p* = 0.036) disruption at baseline and extensive DRIL (*t* = 2.922, *p* = 0.004) were associated with nonresponse defined as < 3 lines of BCVA improvement at 6 months after the final anti‐VEGF injection. A multivariate logistic regression analysis indicated that, for an N1 implicit time of R1, each 1‐ms reduction in N1 implicit time for Ring 1 corresponded to an adjusted odds ratio of 1.252 for favorable visual response (95% CI [1.101, 1.422], *p* = 0.001) after anti‐VEGF treatment. The results were the same for an N1 implicit time of R2 (1.111, 95% CI [1.028, 1.201], *p* = 0.008), a P1 implicit time of R1 (1.049, 95% CI [0.998, 1.102], *p* = 0.029), and a P1 implicit time of R2 (1.105, 95% CI [1.012, 1.206], *p* = 0.025).

**Conclusions:**

Baseline SD‐OCT and mfERG biomarkers were significantly associated with anti‐VEGF treatment outcomes in this DME cohort. Shorter N1 and P1 implicit times in the first and second mfERG rings were correlated with favorable treatment responses, while extensive disruption of ELM and EZ, severe DRIL, and the presence of tractional ERM were linked to poorer anti‐VEGF responses. These structural and functional biomarkers may offer potential prognostic reference value for evaluating treatment prognosis. Further large‐sample and prospective studies are still required to validate their clinical applicability and routine use in guiding therapeutic decision‐making.

## 1. Introduction

Increasing clinical use of anti‐vascular endothelial growth factor (anti‐VEGF) therapy has improved visual outcomes for patients with diabetic macular edema (DME). Nguyen et al. were the first to report the results of this treatment in two 24‐month Phase III randomized studies (RISE and RIDE). They found that ranibizumab rapidly and sustainably improves vision, reduces the risk of further vision loss, and improves macular edema in patients with DME, with low rates of ocular and nonocular harm [1].

Most subjects in the study of Nguyen et al. demonstrated improved two‐line visual acuity (VA) on the Early Treatment Diabetic Retinopathy Study (ETDRS) visual acuity chart within two years. However, they used differentiated validation, and some patients who had received anti‐VEGF treatment in three consecutive months responded poorly, showing improvements of less than five letters in best‐corrected visual acuity (BCVA) [2, 3]. Randomized clinical trials have reported frequencies of nonresponse to anti‐VEGF treatment of 20%–65% [4].

Several studies have examined resistance to anti‐VEGF treatment; most of these focused on ultrastructural factors reflected in spectral domain optical coherence tomography (SD‐OCT). Patients with integrated external limiting membrane (ELM) and ellipsoid zone (EZ) at baseline have excellent visual outcomes [5–7]. Furthermore, Velaga et al. evaluated the impact of the inner retinal layers on the outcomes of anti‐VEGF treatment in a longitudinal prospective study. They show that decreases in the area and maximum extent of disorganization of the retinal inner layers (DRIL) is negatively correlated with increases in BCVA 24 weeks after a loading dose of anti‐VEGF treatment [8]. Most prior studies only evaluated SD‐OCT structural predictors for anti‐VEGF response in DME, while few combined macular multifocal electroretinography (mfERG) functional biomarkers to jointly predict treatment outcomes, which is the core novelty of our present work.

However, the possible correlations of baseline macular functional biomarkers with the outcomes of anti‐VEGF treatment have not yet been elucidated. No studies have been conducted in previous reports. Our study aims to determine the predictors of poor response for anti‐VEGF treatment.

## 2. Materials and Methods

This retrospective study was conducted at Hebei Eye Hospital between December 2022 and January 2024. It was approved by the Institutional Review Board of Hebei Eye Hospital and adhered to the Declaration of Helsinki. All participants gave written informed consent at the time of recruitment.

### 2.1. Inclusion and Exclusion Criteria

All subjects were recruited at Hebei Eye Hospital consecutively. The inclusion criteria were as follows: (1) DME patients who had received 3 anti‐VEGF treatments consecutively at 1‐month intervals; (2) patients with Type 2 diabetes mellitus under endocrinologist care for no less than 3 months; (3) patients identified the presence of DME by fundus fluorescein angiography (FFA); (4) patients with a central subfield thickness (CST) of ≥ 320 mm for men and ≥ 305 mm for women on Heidelberg Spectralis OCT; (5) BCVA from 20/200 to 20/40 using the ETDRS standard charts; (6) patients who had not received cataract surgery within 6 months before the first intravitreal injection; and (7) patients who had not received therapy for DME within 6 months before the first intravitreal injection. The exclusion criteria were as follows: (1) prior posterior segment treatments, including laser photocoagulation and pars plana vitrectomy; (2) corneal or lens opacity that could compromise OCT and multifocal electroretinography tests; (3) retinal abnormalities other than DME, including age‐related macular degeneration, hypertensive retinopathy, or vascular occlusion; prior or active inflammatory ocular pathologies, such as uveitis; (4) failure to comply with the study visit schedule, insufficient cooperation in electrophysiological care, and withdrawal of consent; and (5) any related complications from intravitreal injections, such as endophthalmitis, retinal detachment or intravitreal hemorrhage, and any systemic complications associated with the treatment agent [9].

General characteristics, including gender, age, renal function (evaluated by estimated glomerular filtration rate eGFR), history or current diagnosis of hypertension (systolic > 120 mmHg, diastolic > 80 mmHg) were assessed at baseline. The glycosylated hemoglobin (HbA1c) was also recorded. Comprehensive ophthalmological examinations included intraocular pressure, slit‐lamp biomicroscopy, color fundus photos, and FFA. BCVA was measured using Snellen charts. The Snellen VA measurements were converted to logarithm of the minimum angle of resolution (logMAR) units so that statistical analysis could be performed.

The structural and functional biomarkers were obtained from all patients under the same conditions using SD‐OCT (Spectralis, Heidelberg, Germany) and multifocal electroretinography (mfERG) examination system (RETI‐Port/Scan 21, Roland, Germany), respectively. The scan passing through the fovea was selected, for seven scans in total (spaced 120 μm apart, encompassing a vertical height of 0.72 mm). Seven 120‐μm‐spaced scans centered on the fovea were analyzed rather than a single transfoveal slice to capture the full extent of macular layer damage. The horizontal span of ELM, EZ, and DRIL disruption was measured on each scan, and mean values across seven slices were calculated to minimize sampling bias. All analyses were restricted to a 1000‐μm fovea‐centered region, ensuring foveal pathology was the core focus of severity grading [10]. Based on these OCT images, DME was classified into three groups: diffuse, cystoid, and mixed. Eyes with subretinal fluid (SRF) were also recorded [11]. Vitreoretinal interface problems were evaluated as the presence of ERM with and without a traction component. ERM was categorized into nontractional ERM and tractional ERM (T‐ERM). Nontractional ERM presented as flat thin hyperreflective membranes without retinal deformation, while T‐ERM was defined as thickened epiretinal tissue generating tangential or vertical traction, causing retinal folds and distorted foveal morphology on SD‐OCT. All scans were graded by a masked physician to separate patients into subgroups with isolated ERM or T‐ERM. The extent of DRIL, EZ, and ELM disruptions were graded as none, mild (< 250 μm), moderate (250–500 μm), and extensive [6] (≥ 500 μm) based on the average horizontal measurement from the seven scans. According to the Writing Committee for the Diabetic Retinopathy Clinical Research Network (DRCR) in 2015 [12], the treatment eligibility threshold was set at a CST of ≥ 320 mm for men or ≥ 305 mm for women on Heidelberg Spectralis OCT. The SD‐OCT and mfERG examinations were completed by two alternative technicians (Ziqi Zhao and Wei Ren) who were masked to patient identities, and the biomarkers obtained from SD‐OCT and mfERG were assessed by a qualified physician (Lina Lv) following the protocol of DRCR and ISCEV (International Society for Clinical Electrophysiology of Vision).

The mfERG was conducted in photopic conditions with the pupil of the study eye dilated and was recorded using corneal contact lens electrodes. The distance from the chinrest to the stimulation screen was 33 cm, and correction lenses (+3 diopter) were added to patients’ own refractive errors. The stimulations were displayed on a cathode ray tube (CRT) 19″ monitor with luminance at 220 cd/m2. The study eye was stimulated with 8 runs of 61 hexagonal elements, covering 54° of the retina (27°radius from fixation point); these stimulation units increased with the increase in eccentricity [13, 14]. The first‐kernel responses in each ring of N1 and P1 wave amplitudes and implicit times were documented according to the concentric ring analysis. The indices of the first and second rings at an eccentricity of 0°–5°, which corresponded to the fovea showing on the OCT, were calculated for statistics. Mean amplitude (in nanovolts/degrees squared nv/deg^2^) and implicit time (in milliseconds) were recorded.

Each patient received 3 anti‐VEGF injections at 1‐month intervals in the operating room. All participants received three consecutive monthly intravitreal injections of conbercept as the standardized loading regimen. No additional anti‐VEGF injections, laser therapy, or other ocular interventions were administered to any patient during the 6‐month follow‐up after the third injection. The anti‐VEGF drug used in our research was conbercept (KH902), a humanized soluble VEGF receptor (VEGFR) protein comprising extracellular domain 2 of VEGFR‐1, and extracellular domains 3 and 4 of VEGFR2 [15]. After injection, topical antibiotic therapy was given 4 times a day for 7 days. The day after injection, all patients underwent slit‐lamp anterior segment examination and posterior segment examination with a 90‐diopter lens. Intraocular pressure measured using Goldmann applanation tonometry was also documented. Routine BCVA measurement was performed at 1 month after the last injection for clinical follow‐up. The primary study endpoint was set at 6 months after the final anti‐VEGF injection, and BCVA at this 6‐month time point was used for grouping of treatment response and nonresponse. The primary endpoint was BCVA assessed at 6 months after the last injection. Unlike most short‐term follow‐up designs, this 6‐month time point was chosen to reflect stable sustained visual function rather than transient edema relief. No supplementary ocular treatments were provided during the 6‐month follow‐up period to avoid confounding effects on final visual outcomes.

Subjects were divided into two groups based on the BCVA outcomes 6 months after anti‐VEGF treatment. The response group (RG) was defined as having three or more lines of improvement in logMAR VA, and the nonresponse group (NRG) was defined as having less than three lines of improvement [6]. During the follow‐up interval from 1 month to 6 months after the third anti‐VEGF injection, no additional intravitreal anti‐VEGF agents, retinal laser therapy, cataract surgery, or other ocular interventions related to DME were given to all enrolled patients, to eliminate the confounding effect of additional treatment on final visual outcomes. This binary categorization was adopted for clinical practicability, while it should be acknowledged that such dichotomization may bring ceiling effect and cannot fully reflect the continuous changes of ΔlogMAR. Severity of diabetic retinopathy (DR) was evaluated using the Diabetic Retinopathy Severity Scale (DRSS) based on detailed evaluation of baseline FFA or fundus photographs [16].

### 2.2. Statistical Analysis

Statistical analysis was performed using SPSS version 25.0 (IBM, Armonk, NY, USA). Continuous variables in the measurement data were expressed as means and standard deviations. Differences between groups were analyzed using the independent samples *t*‐test. The Shapiro–Wilk test was used to assess normality. Categorical variables were described as percentages and compared via the χ^2^ test or Fisher exact probability test. Baseline SD‐OCT biomakers were transferred through dummy variable processing, and linear regression analysis was performed to determine anti‐VEGF treatment response. For multivariate analyses, different regression models were selected according to research purpose and outcome type. The primary study outcome was dichotomized anti‐VEGF treatment response (favorable versus nonresponse), defined as ≥ 3 lines of logMAR VA improvement at 6‐month follow‐up. Linear regression was applied as a supplementary analysis to assess the associations between baseline SD‐OCT biomarkers and continuous changes in logMAR BCVA from baseline to 6‐month follow‐up. Multivariate logistic regression was performed to explore the relationships between baseline mfERG parameters and the primary binary treatment response outcome. For the multivariate logistic regression model, a combined clinical‐driven and statistical variable selection strategy was adopted. Clinically recognized confounding factors, including baseline BCVA (logMAR), age, hypertension, HbA1c, and DR stage, were directly incorporated into the final regression model regardless of univariate *p* values, to sufficiently control confounding effects, avoid omitting clinically important covariates, and improve model stability. For the core mfERG biomarkers, only indices with significant intergroup differences in univariate analysis (*p* < 0.05) were further entered into the multivariate model together with the above fixed confounding covariates. To mitigate the risk of model overfitting given the limited sample size, a stringent variable selection strategy was applied. Only five clinically essential confounders were forced into the model regardless of univariate p‐values, and only mfERG parameters with significant intergroup differences (*p* < 0.05) were further included as predictive variables. The final model contained 9 predictors, satisfying the standard 10 events per predictor (EPV) criterion with 39 nonresponse events. Sensitivity analyses were performed by iteratively omitting single mfERG predictors to confirm the stability of effect estimates. To avoid the impact of binocular homogeneity, data from the right eye were used for intergroup comparisons in patients with bilateral eyes influenced. A *p*‐value of less than 0.05 was considered significant in all the analyses. Bonferroni correction was performed for multiple intergroup comparisons of SD‐OCT biomarkers. With 12 independent subgroup comparisons, the adjusted significance cutoff was set at α = 0.0042. All intergroup p‐values for OCT biomarkers were less than 0.001, which remained significant after adjustment. No multiple comparison correction was applied to other statistical tests including demographic comparisons, mfERG *t*‐tests, linear regression, and multivariate logistic regression.

## 3. Results

In total, 87 patients completed the loading dose and were followed for 6 months. A total of 48 subjects were included in the RG, and 39 were included in the NRG. The total number of anti‐VEGF injections and injection intervals were identical for all subjects in both the RG (*n* = 48) and NRG (*n* = 39). A comparison of the general baseline demographics of the two groups is provided in Table 1.

**TABLE 1:** Comparison of the general baseline demographics of the two groups.

Demographic variable	Response group (RG)	Nonresponse group (NRG)	*x* ^2^ (t, z)	*p*
Male/female (*n*)	27/21	19/20	0.49	0.484
Age (M ± SD, years)	61.96 ± 8.03	61.91 ± 7.69	0.026	0.979
Hypertension *n* (%)	22 (45.8%)	19 (48.7%)	0.072	0.789
Chronic renal failure *n* (%)	12 (25%)	11 (28.2%)	0.114	0.736
HbA1c (M ± SD, mmol/L)	8.13 ± 1.12	8.01 ± 1.07	0.546	0.586
VA (median IQR, logMAR)	0.81 (0.45, 1.28)	0.85 (0.55, 1.39)	−1.016	0.310
DR stage (median IQR)	4 (3, 5)	4 (2, 5)	−0.607	0.544

*Note:* The patients with eGFR < 60 mL/min/1.73 m^2^ were diagnosed chronic renal failure.

Abbreviations: DR, diabetic retinopathy; logMAR, logarithm of minimum angle of resolution; VA, visual acuity.

No discrepancies in the general demographics distribution between the RG and NRG were found. VA and DR stages, which might have influenced therapeutic outcomes, were also compared, but the differences were not statistically significant.

Macular edema was classified into diffuse, cystoid, or mixed types based on SD‐OCT images, and there were no statistically significant differences in anti‐VEGF treatment responses among DME subtypes (*χ* ^2^ = 1.94, *p* = 0.379). Although baseline CST was thicker in the NRG, no statistically significant difference was found between the groups (z = −0.166, *p* = 0.868). Similarly, patients with SRF or nontractional ERM initially had comparable anti‐VEGF responses (*χ* ^2^ = 0.530, *p* = 0.467; *χ* ^2^ = 1.056, *p* = 0.304, respectively). However, patients with tractional ERM (T‐ERM) were more prone to have anti‐VEGF resistance (*χ* ^2^ = 27.45, *p* < 0.001).

There were tremendous differences between the two groups in terms of ELM disruption, DRIL, and EZ disruption (*χ* ^2^ = 27.207, *p* < 0.001; *χ* ^2^ = 27.902, *p* < 0.001; *χ* ^2^ = 17.583, *p* < 0.001, respectively). DME patients showed apparently poor outcomes with baseline extensive inner and outer layer disruption, which were exhibited on SD‐OCT. A comparison of the two groups’ baseline OCT biomarkers is shown in Table 2.

**TABLE 2:** Comparison of the baseline OCT biomarkers of the two groups after anti‐VEGF treatment.

Biomarker	RG	NRG	*X* ^2^ (z)	*p*
CST (median IQR, μm)	472 (389,590)	532 (337, 680)	−0.166	0.868
ERM *n* (%)	23 (47.9%)	23 (59%)	1.056	0.304
T‐ERM *n* (%)	5 (10.4%)	25 (64.1%)	27.45	< 0.001
SRF *n* (%)	7 (14.6%)	8 (20.5%)	0.530	0.467
DME type			1.94	0.379
	Diffuse 29 (60.4%)	19 (48.7%)		
	Cystoid 13 (27.1%)	11 (28.2%)		
	Mixed 6 (12.5%)	9 (23.1%)		
ELM disruption			27.207	< 0.001
None	0 4 (8.3%)	2 (5.1%)		
Mild	< 250 μm 22 (45.9%)	3 (7.7%)		
Moderate	250–500 μm 17 (35.4%)	12 (30.8%)		
Extensive	> 500 μm 5 (10.4%)	22 (56.4%)		
DRIL			27.902	< 0.001
None	0 5 (10.4%)	4 (10.3%)		
Mild	< 250 μm 24 (50%)	7 (18%)		
Moderate	250–500 μm 15 (31.3%)	5 (12.8%)		
Extensive	> 500 μm 4 (8.3%)	23 (58.9%)		
EZ disruption			17.583	< 0.001
None	0 4 (8.3%)	3 (7.7%)		
Mild	< 250 μm 27 (56.3%)	7 (18%)		
Moderate	250–500 μm 7 (14.6%)	5 (12.8%)		
Extensive	> 500 μm 10 (20.8%)	24 (61.5%)		

*Note:* Bonferroni correction was applied for multiple OCT subgroup comparisons; adjusted significance threshold = 0.0042. All intergroup differences retained statistical significance after correction.

Abbreviations: CST, central subfield thickness; DME, diabetic macular edema; DRIL, disorganization of retinal inner layers; ELM, external limiting membrane; EZ, ellipsoid zone; SRF, subretinal fluid; T‐ERM, tractional epiretinal membrane.

**FIGURE 1:**
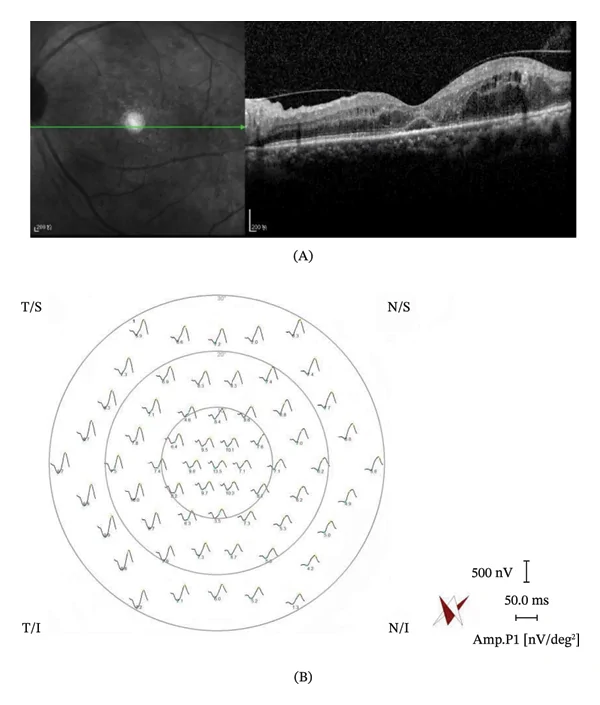
(A) Cystoid DME with subfoveal fluid, extensive ELM/EZ disruption, widespread disorganized retinal inner layers (DRIL), and tractional epiretinal membrane (T‐ERM, marked by retinal folds and upward retinal traction) from a patient in the nonresponse group. (B) Trace array of the same patient in the nonresponse group.

We conducted a linear regression analysis after dummy variable processing to assess the relationships among different grades of disturbances on SD‐OCT and visual outcomes. It revealed that subjects with extensive ELM disruption, DRIL, and EZ disruption at baseline manifested resistant responses to anti‐VEGF treatment (*p* = 0.004, *p* = 0.004, and *p* = 0.036, respectively; see Table 3). Figure 1A shows a patient who presented with extensive DRIL, ELM, and EZ disruption on SD‐OCT. Figure 1B presents the trace array of mfERG from the same subject.

**TABLE 3:** Linear regression analysis results for baseline SD‐OCT biomarkers and anti‐VEGF treatment efficacy.

Biomarkers	*B* [95% CI]	*t*	*p*
ELM disruption			
None	0.023 (−0.211, 0.257)	0.191	0.849
Mild	−0.303 (−0.545, −0.061)	−2.469	0.104
Moderate	−0.023 (−0.257, 0.211)	−0.191	0.849
Extensive	0.351 (0.114, 0.589)	2.914	0.004
DRIL			
None	−0.061 (−0.267, 0.144)	−0.588	0.557
Mild	−0.219 (−0.501, 0.064)	−1.525	0.128
Moderate	−0.194 (−0.477, 0.089)	−1.354	0.177
Extensive	0.407 (0.133, 0.682)	2.922	0.004
EZ disruption			
None	−0.245 (−0.474, −0.016)	−2.112	0.063
Mild	−0.244 (−0.476, −0.012)	−2.075	0.309
Moderate	0.045 (−0.217, 0.308)	0.342	0.733
Extensive	0.245 (0.016, 0.474)	2.112	0.036

We also evaluated the biomarkers of the first and second rings of mfERG, which corresponded to the fovea on SD‐OCT. The two groups yielded significant functional differences. Interestingly, the differences of N1 and P1 implicit times in the first and second rings between the two groups were statistically significant (*t* = −3.593, *p* < 0.001; *t* = −2.583, *p* = 0.011; *t* = −2.748, *p* = 0.006; *t* = −3.953, *p* < 0.001, respectively). However, the differences between their N1 and P1 amplitudes in the first and second rings were not statistically significant (*t* = −0.192, *p* = 0.848; *t* = −1.408, *p* = 0.160; *t* = −0.065, *p* = 0.948; *t* = −0.724, *p* = 0.470, respectively). The results are shown in Table 4.

**TABLE 4:** Baseline biomarkers of N1 and P1 waves in the first and second rings of mfERG.

Biomarker	RG	NRG	*t*	*p*
N1 implicit time in R1 (ms)	24.86 ± 1.96	26.03 ± 3.00	−3.593	< 0.001
P1 implicit time in R1 (ms)	43.83 ± 6.81	45.77 ± 4.40	−2.583	0.011
N1 implicit time in R2 (ms)	29.55 ± 3.16	30.85 ± 4.14	−2.748	0.006
P1 implicit time in R2 (ms)	46.26 ± 3.71	48.00 ± 3.03	−3.953	< 0.001
N1 amplitude in R1 (nv/deg^2^)	11.53 ± 2.75	11.45 ± 2.95	−0.192	0.848
P1 amplitude in R1 (nv/deg^2^)	34.67 ± 6.93	33.44 ± 6.60	−1.408	0.160
N1 amplitude in R2 (nv/deg^2^)	7.79 ± 2.15	7.77 ± 2.23	−0.065	0.948
P1 amplitude in R2 (nv/deg^2^)	18.74 ± 3.98	18.23 ± 6.36	−0.724	0.470

*Note:* R1, first ring; R2, second ring.

**FIGURE 2:**
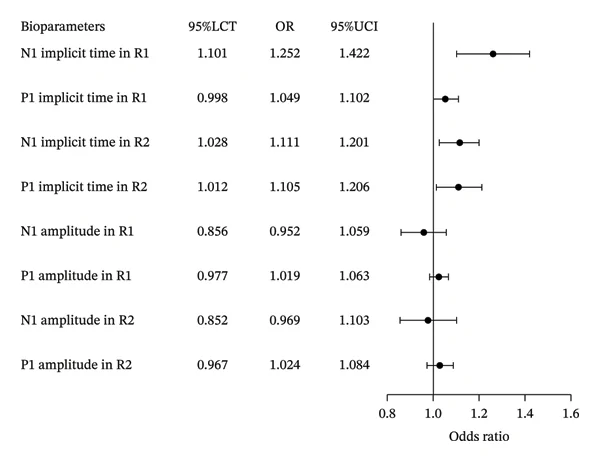
Forest map of baseline mfERG biomarkers associated with BCVA outcomes after anti‐VEGF treatment. Note: Variables with *p* > 0.05 in the logistic regression analysis of the association between baseline mfERG biomarkers and BCVA outcomes are also represented in the figure.

Logistic regression analysis findings for baseline mfERG biomarkers associated with improved BCVA consequences after anti‐VEGF treatment are summarized in Table 5. After adjustment for baseline BCVA and other clinical confounding factors, the significant prognostic role of N1 and P1 implicit times in the first and second mfERG rings remained independent, indicating that the associations were not attributed to differences in baseline visual acuity alone. Variables showing an association in the univariate model (*p* < 0.05) were included in the multivariate model. More shortened implicit times of N1 and P1 waves of the first and second rings at the beginning were prominent predictors for efficacious outcomes of anti‐VEGF treatment (*p* = 0.001, *p* = 0.029, *p* = 0.008, and *p* = 0.025, respectively). Figure 2 shows that baseline mfERG biomarkers associated with BCVA outcomes after anti‐VEGF treatment and N1 and P1 amplitude in R1 and R2 were not statistically significant.

**TABLE 5:** Logistic regression analysis results showing the association between baseline biomarkers of N1 and P1 waves in the first and second rings of mfERG and anti‐VEGF treatment efficacy.

Biomarker	Univariate		Multivariate	
	Odds ratio (95% CI)	*p*	Odds ratio (95% CI)	*p*
N1 implicit time in R1 (per 1 ms shortened)	1.202 (1.079, 1.338)	0.001	1.252 (1.101, 1.422)	0.001
P1 implicit time in R1 (per 1 ms shortened)	1.062 (1.015, 1.112)	0.010	1.049 (0.998, 1.102)	0.029
N1 implicit time in R2 (per 1 ms shortened)	1.099 (1.025, 1.179)	0.008	1.111 (1.028, 1.201)	0.008
P1 implicit time in R2 (per 1 ms shortened)	1.166 (1.076, 1.263)	< 0.001	1.105 (1.012, 1.206)	0.025

*Note:* Variables with *p* < 0.05 in the univariate model were entered into a multivariate model; R1, first ring; R2, second ring.

Abbreviation: CI = confidence interval.

## 4. Discussion

According to the ETDRS protocol, anti‐VEGF treatment has been the first‐line treatment for DME for the last decade [1]. However, many subjects do not show a favorable visual outcome after a loading dose treatment. Recent studies have demonstrated that although 55.6% of affected eyes achieved a BCVA gain of greater than or equal to five letters, 29.6% remained stable, and 14.8% exhibited a deterioration in BCVA of more than five letters [3, 17].

Previous studies have confirmed the baseline risk factors identified on SD‐OCT, which were correlated with poor response [6–9]. However, few studies have explored the association between baseline macular functional biomarkers derived from mfERG and anti‐VEGF treatment outcomes in DME patients, and this clinical issue remains insufficiently investigated. Our findings suggested that baseline N1 and P1 implicit times in the first and second rings of mfERG played a key role in anti‐VEGF treatment in DME patients. Moreover, similar to the research of Koc et al. [6], our findings indicate that eyes with T‐ERM, extensive ELM, EZ disruption, and extensive DRIL revealed on SD‐OCT initially were nonresponsive to anti‐VEGF treatment.

Notably, baseline BCVA was fully adjusted in the multivariate logistic regression model in our study. Even after controlling for baseline visual acuity and other clinical covariates, prolonged baseline N1 and P1 implicit times still independently associated with poor anti‐VEGF treatment response. This further demonstrates that mfERG neurophysiological biomarkers provide additional key value beyond baseline BCVA.

The N1 wave is generated by photoreceptors [18]. Eyes with extensive EZ disruption on SD‐OCT showed severe photoreceptor damage, with potential loss of function in the inner and outer segments. Additionally, the time required to convert light signals into electronic signals was prolonged, and the neural impulse amplitude was reduced [19]. In our study, patients with extensive EZ disruption showed delayed implicit time and decreased amplitude of the N1 wave in the first and second rings, which corresponds to the foveal region on SD‐OCT.

Compared to the RG, the N1 implicit time in R1 and R2 of the NRG was longer, and the differences were statistically significant (*p* < 0.001 and *p* = 0.006, respectively). However, the differences in the amplitudes of N1 in R1 and R2 between the RG and NRG were not statistically significant (*p* = 0.848 and *p* = 0.948, respectively). Compared to local implicit time, local amplitude had higher intersubject variability. This resulted in smaller confidence intervals than those obtained for local mfERG amplitudes, which thus require a larger relative deviation from normal to reach statistical significance [20].

There was a general consensus that the P1 wave was generated by Müller and bipolar cells. The ELM disruption and DRIL were dramatic at baseline in the NRG, which indicated that the destruction of inner retinal layers was prominent. Moreover, Müller and bipolar cells were disturbed and the inner layer was disturbed by T‐ERM, and the structural and functional of macular were damaged. As a consequence, the amplitude and implicit time of the P1 wave was influenced, and the alternations were comparable to the N1 wave. Compared to the RG, the implicit time of P1 in R1 and R2 of the NRG was longer, and the differences were statistically significant (*p* = 0.011 and *p* < 0.001, respectively). However, the differences in the amplitudes of P1 in R1 and R2 between the RG and NRG were not statistically significant (*p* = 0.160 and *p* = 0.470, respectively).

A previous histopathological study showed that DME usually develops at the inner layer and causes severe necrosis in Müller and bipolar cells. Damage to the inner layer was more pronounced than that to the outer layer [21, 22]. The results obtained in our research differed from this previous study’s results. This was likely due to different distribution in DME subtypes and the proportion of the inner and outer layers’ disruption might be different. Our study provided more explicit classifications of DME—as well as ELM, EZ, and DRIL—than previous studies.

It has been demonstrated that the implicit times of N1 and P1 in the first and second rings were highly correlated with the outcome in multivariate logistic regression analysis. Subjects with shorter implicit times were more likely to achieve ideal visual outcomes. For N1 implicit times in R1, each 1‐ms shortening was associated with a 1.252‐fold increase in the odds of achieving a favorable anti‐VEGF treatment outcome (95% CI [1.101, 1.422], *p* = 0.001). Identical results were obtained for N1 implicit times in R2 (1.111, 95% CI [1.028, 1.201], *p* = 0.008), P1 implicit times in R1 (1.049, 95% CI [0.998, 1.102], *p* = 0.029), and P1 implicit times in R2 (1.105, 95% CI [1.012, 1.206], *p* = 0.025). Odds ratios and 95% confidence intervals are shown in Table 5 and Figure 2.

We also investigated the structural differences between two groups at baseline. Extensive ELM disruption was correlated with poor response (*t* = 2.914, *p* = 0.004). The ELM is composed of a network of adherens junctions between Müller cells that maintain the integrity of the photoreceptors’ inner segments by forming a diffusion barrier between the subretinal space and the inner retina [23, 24]. The Müller cells control the composition of the extracellular fluid and regulate the tightness of the blood–retinal barrier [25–27]. Extensive ELM disruption leads to vascular telangiectasia, breakdown of the blood–retinal barrier, and intraretinal neovascularization. Although the integrity of the ELM is partially restored after anti‐VEGF treatment, its function in supporting the homeostatic and metabolism of retinal neurons cannot be fully restored [28].

It is controversial whether the unfavorable outcomes were associated with EZ disruption and DRIL after anti‐VEGF treatment [29, 30]. In our research, extensive EZ disruption and DRIL were significantly associated with anti‐VEGF outcomes (*t* = 2.112, *p* = 0.036; *t* = 2.922, *p* = 0.004, respectively). EZ was the second hyperreflective band in SD‐OCT B‐scan images of the outer retina. Additionally, EZ played an important role in the outer barrier of the retina. Extensive EZ disruption might lead to severe accumulation of SRF and exacerbate photoreceptor damage. Although SRF is eliminated after intravitreal anti‐VEGF drugs, the function of photoreceptor might not improve. During long‐term edema, the photoreceptor became necrotic. Nevertheless, residual EZ interruptions visible on SD‐OCT are associated with worse visual outcomes [31, 32].

DRIL is a sign of macular capillary nonperfusion and is associated with impaired retinal function [33, 34]. We found that extensive DRIL was an SD‐OCT biomarker that was correlated with a negative anti‐VEGF treatment response. Dodo et al. verified that DRIL severity indicates damage to the pathways that carry visual data from photoreceptors to ganglion cells [35]. In addition, the combination of EZ disruption and DRIL is associated with a further significant worsening of VA [36].

Under chronic hyperglycemia, accumulation of advanced glycosylation end‐products causes abnormal collagen cross‐linking, which leads to stronger adhesions between the posterior vitreous and inner limiting membrane [37]. Furthermore, anti‐VEGF agents tend to cause an angiofibrotic shift [38], which can generate traction forces on the retina and potentially accelerate the formation of T‐ERM. The tangential and vertical traction forces of T‐ERM can aggravate macular edema and exacerbate abnormality of the inner retina, especially in extensive T‐ERM [39]. In our study, there was a statistically significant difference in the distribution of T‐ERM between the RG and NRG (*χ* ^2^ = 27.45, *p* < 0.001).

This study has some limitations. The sample size was relatively small. Inherent bias, limited generalizability, and weakness in establishing causality may have arisen due to the retrospective design. Macular perfusion status was not evaluated via OCTA, which may significantly influence retina function and anti‐VEGF outcomes. We assessed BCVA six months post‐treatment; however, a minority of subjects may show a delayed response to the loading dose rather than nonresponse to anti‐VEGF treatment. Furthermore, functional and structural recovery of DME may be asymmetrical: although the biomarkers of SD‐OCT were restored to normal levels, BCVA did not reach an ideal level. Dichotomizing treatment response based on categorical BCVA line improvement is clinically interpretable but inevitably introduces potential ceiling effects and information loss, as it cannot fully reflect continuous ΔlogMAR visual acuity changes. We classified treatment response by a binary cutoff of ≥ 3 lines BCVA improvement, which cannot fully capture continuous visual changes and may cause information loss. To alleviate this bias, we supplemented linear regression using continuous ΔlogMAR values. Another limitation lies in the relatively small total sample size (87 patients) for the multivariate logistic regression model. Although we strictly restricted the number of predictive variables following the 10‐events‐per‐predictor rule and conducted sensitivity tests to verify model stability, the limited cohort scale may still introduce subtle model instability and restrict the generalizability of our odds ratio estimates. Large‐scale prospective cohorts with larger event numbers are required to validate the independent prognostic value of mfERG implicit time biomarkers and further simplify predictive models for clinical application. Future studies relying on continuous visual endpoints will help confirm our results. The present findings should be interpreted as retrospective exploratory prognostic associations, rather than formal predictive modeling or independent validation results. Additionally, some unmeasured or incompletely quantified clinical and systemic confounders may still influence treatment outcomes and the interpretation of our associations. Further large‐sample prospective studies adopting continuous visual endpoints and more comprehensive confounding adjustment are warranted to validate and extend our findings. Another limitation was that we evaluated only scans within the fovea‐centered 1000‐μm area for each eye in our study, which could not completely include the whole edematous macula. Additionally, although volume scans might have been more accurate, they were not implemented.

## 5. Conclusion

Baseline SD‐OCT and mfERG biomarkers were significantly associated with anti‐VEGF treatment outcomes in this DME cohort. Shorter N1 and P1 implicit times in the first and second mfERG rings were correlated with favorable treatment responses, while extensive disruption of ELM and EZ, severe DRIL, and the presence of tractional ERM were linked to poorer anti‐VEGF responses. These structural and functional biomarkers may offer potential prognostic reference value for evaluating treatment prognosis. Further large‐sample and prospective studies are still required to validate their clinical applicability and routine use in guiding therapeutic decision‐making.

## Author Contributions

Rongrong Li conceived and designed the experiments. Yi Cai wrote the manuscript. Ziyi Wei, Yong Liu, and Yi Gao performed the data analyses. Lina Lv and Yanan Wang were responsible for the handling of pictures. Zeming Wu collected the data.

## Funding

This study was supported by grants from the Municipal Science and Technology Plan Project of Xingtai City, Hebei Province, No. 2025ZC058.

## Disclosure

The authors read and approved the final manuscript.

## Ethics Statement

This study was approved by the Ethics Committee of Hebei Eye Hospital and was performed in adherence to the principles of the Declaration of Helsinki. Written informed consent was obtained from all participants.

## Consent

Please see the Ethics Statement.

## Conflicts of Interest

The authors declare no conflicts of interest.

## Data Availability

The datasets used and/or analyzed during the current study are available from the corresponding author upon reasonable request.
